# Activation of PKC triggers rescue of NPC1 patient specific iPSC derived glial cells from gliosis

**DOI:** 10.1186/s13023-017-0697-y

**Published:** 2017-08-25

**Authors:** Franziska Peter, Sebastian Rost, Arndt Rolfs, Moritz J. Frech

**Affiliations:** Albrecht-Kossel-Institute for Neuroregeneration (AKos), University Medicine Rostock, Gehlsheimer Straße 20, 18147 Rostock, Germany

**Keywords:** NPC1, IPSCs, PKC, Vimentin, GFAP

## Abstract

**Background:**

Niemann-Pick disease Type C1 (NPC1) is a rare progressive neurodegenerative disorder caused by mutations in the *NPC1* gene. The pathological mechanisms, underlying NPC1 are not yet completely understood. Especially the contribution of glial cells and gliosis to the progression of NPC1, are controversially discussed. As an analysis of affected cells is unfeasible in NPC1-patients, we recently developed an in vitro model system, based on cells derived from NPC1-patient specific iPSCs. Here, we asked if this model system recapitulates gliosis, observed in non-human model systems and NPC1 patient post mortem biopsies. We determined the amount of reactive astrocytes and the regulation of the intermediate filaments GFAP and vimentin, all indicating gliosis. Furthermore, we were interested in the assembly and phosphorylation of these intermediate filaments and finally the impact of the activation of protein kinase C (PKC), which is described to ameliorate the pathogenic phenotype of NPC1-deficient fibroblasts, including hypo-phosphorylation of vimentin and cholesterol accumulation.

**Methods:**

We analysed glial cells derived from NPC1 patient specific induced pluripotent stem cells, carrying different NPC1 mutations. The amount of reactive astrocytes was determined by means of immuncytochemical stainings and FACS-analysis. Semi-quantitative western blot was used to determine the amount of phosphorylated GFAP and vimentin. Cholesterol accumulation was analysed by Filipin staining and quantified by Amplex Red Assay. U18666A was used to induce NPC1 phenotype in unaffected cells of the control cell line. Phorbol 12-myristate 13-acetate (PMA) was used to activate PKC.

**Results:**

Immunocytochemical detection of GFAP, vimentin and Ki67 revealed that *NPC1* mutant glial cells undergo gliosis. We found hypo-phosphorylation of the intermediate filaments GFAP and vimentin and alterations in the assembly of these intermediate filaments in *NPC1* mutant cells. The application of U18666A induced not only NPC1 phenotypical accumulation of cholesterol, but characteristics of gliosis in glial cells derived from unaffected control cells. The application of phorbol 12-myristate 13-acetate, an activator of protein kinase C resulted in a significantly reduced number of reactive astrocytes and further characteristics of gliosis in NPC1-deficient cells. Furthermore, it triggered a restoration of cholesterol amounts to level of control cells.

**Conclusion:**

Our data demonstrate that glial cells derived from NPC1-patient specific iPSCs undergo gliosis. The application of U18666A induced comparable characteristics in un-affected control cells, suggesting that gliosis is triggered by hampered function of NPC1 protein. The activation of protein kinase C induced an amelioration of gliosis, as well as a reduction of cholesterol amount. These results provide further support for the line of evidence that gliosis might not be only a secondary reaction to the loss of neurons, but might be a direct consequence of a reduced PKC activity due to the phenotypical cholesterol accumulation observed in NPC1. In addition, our data support the involvement of PKCs in NPC1 disease pathogenesis and suggest that PKCs may be targeted in future efforts to develop therapeutics for NPC1 disease.

## Background

Niemann-Pick type C1 is a rare lysosomal storage disorder with an incidence of 1.12:100.000 live births [1]. The phenotype of this lipidosis exhibit various symptoms ranging from hepatosplenomegaly, motor dysfunctions, cerebellar ataxia and seizures to dementia, whereby the age of onset varies from early infantile to adult onset forms. The phenotype is based on mutations in either NPC1 (90%) or NPC2 (10%) gene [2]. Due to the defect of the cholesterol transporting NPC1 or NPC2 proteins, located in the lysosomal membrane and lumen, respectively, cholesterol and other lipids like sphingolipids GM2 and GM3 gangliosides accumulate [3]. Recent studies support an interaction of NPC1 and NPC2 mediating the cholesterol efflux from lysosomes, but the exact mechanism is still unexplained [4]. Diagnosis can be performed by biochemical tests like Filipin staining of patient fibroblasts and cholesterol esterification tests, as well as the detection of highly disease specific biomarker, but for final validation a genetic testing is essential [5, 6]. Currently, there is only one, by the European Medicines Agency (EMA) approved, therapy for NPC1 disease, using Miglustat, a reversible inhibitor of glucosylceramide synthase. Miglustat was approved to treat Gaucher disease Type 1, but also improves neurological symptoms in NPC1-patients, whereby the hypothesized mode of action is substrate reduction effect [7, 8]. Further potential therapy approaches are discussed containing the use of cyclodextrins, histone deacetylase inhibitors and chaperons [9, 10]. In regards of the neurological symptoms, like cerebellar ataxia, based on a progressive loss of cerebellar Purkinje cells, the pathological mechanism is not yet understood. Alike, the contribution of gliosis, described in NPC1 patients and different animal models is controversially discussed [11]. Gliosis is not only a ubiquitous event in the central nervous system after any kind of tissue damage [12], but is discussed to be an integral player in neurodegenerative diseases like Alzheimer disease [13], wherein reactive astrocytes can be beneficial as well detrimental for neuroprotection and tissue regeneration [14]. Interestingly, Alzheimer disease and NPC1 share some common features including abnormal cholesterol metabolism, and involvement of amyloid-β and tau pathology [15]. However, gliosis can be elucidated by an increased number of GFAP positive reactive astrocytes [16, 17]. Besides GFAP, an upregulation of other intermediate filaments (IFs) like vimentin and nestin can be observed, as well as an increased number of proliferative cells, demonstrated by Ki67 expression or BrDU incooperation experiments [18, 19]. In regards of NPC1 an increased number of reactive astrocytes and abnormal morphological changes are described in a commonly used NPC1 mouse model [20–22]. NPC1 deficient mice revealed an upregulation of glia cells after 4 weeks of age and astrocytes showed an atypical morphology by less elaborated processes and swollen cell bodies [23]. Gliosis was also shown in human post mortem brain biopsy [24–26]. Although, gliosis is a certainly proved pathological feature of NPC1 the role of glial cells in the progression of the disease is controversially discussed.

Recently we developed a human cell model system, based on induced pluripotent stem cells [27, 28], which was used in this study to elucidate if iPSC derived glial cells resemble gliosis, as a pathogenic hallmark of NPC1. This NPC1 cell model displays typical NPC1 hallmarks, like cholesterol and GM2 accumulations [27, 28], which is in accordance to other studies using comparable approaches [29–31]. However, a description of gliosis in such an in vitro model system is still missing.

Here we investigated gliosis and phosphorylation status of the intermediate filaments GFAP and vimentin in NPC1 patient-specific iPSC-derived glial cells. Intermediate filaments are a non-enzymatic, rod-shaped cytoskeleton component with a length of 8 to 15 nm diameter [32]. IFs are divided into 6 classes whereby class III contains structural proteins like nestin, vimentin and glial fibrillary acidic protein (GFAP). Functions of intermediate filaments are also very variable like cytoarchitecture, cell mobility, mechanical support but also vesicular trafficking and signal modulation [33]. The assembly of intermediate filaments is based on association of monomers whereby this is an assembly cycle consisting of polymerization via dephosphorylation of soluble monomers and depolimerization by phosphorylation of filaments. IFs are phosphorylated by many different kinases like protein kinase C (PKC) or protein kinase A [34–36]. In NPC1 fibroblasts a disturbed vimentin assembly cycle with altered vimentin phosphorylation status and aggregation of vimentin was described [37]. Furthermore, they proved that sphingosine accumulation inhibits protein kinase C, subsequently vimentin was not phosphorylated, the pool of soluble vimentin was decreased and vesicular trafficking was blocked. An increased cholesterol storage in the endosomal/lysosomal system was hypothesized. Consequently activation of PKC, especially PKCε, could increase soluble vimentin and rescued cholesterol esterification effect in NPC1 fibroblasts [37, 38]. Here we demonstrated that NPC1 patient specific iPSC derived glial cells undergo spontaneously gliosis, reflecting a pathological characteristic of NPC1. In addition, we found a disturbed assembly cycle of GFAP and vimentin. Moreover, we were able to rescue the IF phenotype via the activation of PKC by phorbol 12-myristate 13-acetate, also leading to a decreased amount of reactive glial cells, an increased fraction of phosphorylated GFAP and vimentin, and decreased cholesterol accumulation in NPC1 mutant cells.

## Methods

### Cell culture

Human dermal fibroblast cell lines were obtained from Coriell Institute for Medical Research, Camden, USA (NPC1 compound heterozygous mutated: c.1836 A > C [p.E612D], c.1628 delC [p.P543Rfs*20]; GM18436 and NPC1 homozygous mutated: c.3882 T > C [p.I1061T]; GM18453) and Centogene AG, Rostock, Germany (control and NPC1 homozygous mutated: c.1180 T > C [p.Y395H]), respectively. Cells were cultivated in fibroblast medium containing DMEM high glucose, 10% FBS and 1% penicillin/streptomycin. All cells were cultivated at 37 °C in a saturated humidity atmosphere containing 5% CO_2_.

### Differentiation of progenitor cells

The generated neural progenitor cells, differentiated from patient-specific iPS cells [27], were used for 20 passages and seeded at an expansion density of 100,000 cells/cm^2^ on poly-L-ornithine-coated (15 μg/ml; Sigma, Germany)/laminin (10 μg/ml; Trevigen, USA) dishes in proliferation medium containing DMEM, 30% DMEM/F-12, 1X B27, 0.5% penicillin/streptomycin, 20 ng/ml FGF2 (Amsbio, United Kingdom), 20 ng/ml EGF (Peprotech, Germany). For terminal differentiation cells were plated with a density of 45,000 cells/cm^2^ in differentiation medium (DMEM, 30% DMEM/F-12, 1X B27, 0.5% penicillin/streptomycin), which was changed every 4 days over a period of 40 days. For experiments comprising an application of PMA or U18666A, cells were differentiated for 40 days and PMA (10 nM, Cayman Chemicals, USA) or U18666A (1 μg/ml Sigma, Germany) were applied for 24 h or 48 h.

### Immunocytochemistry

Cells were fixed at room temperature for 15 min in 4% paraformaldehyde (PFA), washed with PBS and stored in 0.02% NaN_3_ at 4 °C. Immunocytochemistry was performed for GFAP (1:500, rabbit IgG, Dako, Germany), Vimentin (1:100, mouse IgG, V9, Invitrogen, Germany), Blocking and permeabilization was carried out using 0.3% Triton X-100 and 5% normal goat serum (Dako, Germany) in PBS for 30 min at room temperature. Cells were incubated with primary antibodies for 1 h at room temperature in 1% normal goat serum, followed by three washing steps with PBS. Alexa Fluor 568 (1:500, goat anti-mouse IgG or goat anti-rabbit IgG, Invitrogen, Germany), Alexa Fluor 488 (1:500, goat anti-mouse IgG or goat anti-rabbit IgG, Invitrogen, Germany) were used as secondary antibodies, incubated 1 h at room temperature with 1% normal goat serum in PBS. After washing with PBS cells were stained with DAPI (5 min, 250 ng/ml), washed three times and mounted with Mowiol-DABCO mounting medium. Pictures were taken with a Biozero8000 microscope system (Keyence, Germany) and LSM780 laser scanning microscope (Zeiss, Germany).

### Colocalization image analysis

Colocalization analysis was performed in three independent experiments with digital images taken of randomly chosen fields (*N* = 3, *n* = 12). Mander’s coefficient [39] was used to determine colocalization of GFAP and Vimentin, and of GFAP and Nestin. Mander’s coefficient represents the proportion of overlapping pixels in two channels and it ranges from 0 for no colocalization to 1 for absolute colocalization. The Mander’s coefficient is not dependent on signal intensities of both channels so it can be used even if the intensities differ [39]. Costes automatic threshold quantifies amount of colocalization automatically based on spatial statistics. Therefore, correlation in different regions of the two-dimensional histogram of a two-color image is used to automatically estimate the threshold by identifying the pixel values with a positive Pearson’s correlation coefficient [40]. NIH Image J software [41] plugin JaCoP [42] was used for analysis and determination of colocalization.

### Filipin staining

For Filipin staining cells were fixed at room temperature for 15 min in 4% PFA, washed with PBS, and stored in 0.02% NaN_3_ at 4 °C. Fixated cells were incubated with 0.1 mg/ml Filipin for 45 min. After three washing steps with PBS cells were mounted with Mowiol-DABCO mounting medium. Filipin fluorescence intensities were quantitative determined by taking 10 random pictures of three replicates using a Keyence Biozero 8000 microscope (Keyence, Germany). Analysis of the fluorescence intensities was performed using ImageJ [41] by automatic determination of the threshold and subsequent measurement of the intensities taking area above threshold into account for normalization. Finally, results were normalized to wildtype intensities [43, 44].

### Western blot

Whole cell lysates were obtained by incubation in RIPA-buffer for 15 min on ice. After centrifugation for 20 min at 15,000 xg at 4 °C supernatant was transferred.

Protein concentrations in samples were measured using the bicinchoninic acid assay (Pierce BCA Protein Assay Kit, Thermo Scientific, USA). Samples were boiled for 15 min at 95 °C in 5× Laemmli buffer and separated by SDS-PAGE with precast gels (4–15%, Bio-Rad Laboratories GmbH, Germany). Proteins were transferred to nitrocellulose membrane with a semi-dry blotting system Tans-Blot Turbo (Bio-Rad Laboratories GmbH, Germany). After blotting, membrane was washed with TBS and blocked with TBST, containing 0.1% Tween-20 and 5% milk powder (pH 7.6) or 5% BSA (Roth, Germany), for 1 h at room temperature. Followed by incubation with primary antibodies, Vimentin (1:10,000, rabbit IgG, GeneTex, USA), pSer38-Vimentin (1:1000, GeneTex, USA), GFAP (1:1000, mouse IgG, Cell Signalling Technology, USA) and pSer38-GFAP (1:1000, rabbit IgG, GeneTex, USA) at 4 °C over night and GAPDH (1:10,000, mouse IgG, Abcam, United Kingdom) and β-Actin (1:10,000, mouse IgG, Sigma, Germany) 1 h at room temperature. Western blots were rinsed 3 times with TBST between the usages of several antibodies and incubated with fluorescent dye labelled secondary antibodies, IRDye 680 LT (1:20,000, goat anti-rabbit IgG), IRDye 800 (1:10,000, goat anti-mouse IgG, all LI-COR, Germany), AlexaFluor 680 (1:10,000, goat anti-rat IgG), Invitrogen, Germany). Precision Plus Protein Dual Xtra Standards (Bio-Rad Laboratories GmbH, Germany) was used as a molecular weight marker. Visualization and quantification was performed with Odyssey Infrared Imaging System (LI-COR, Germany). Expression of glyceraldehyde 3-phosphate dehydrogenase (GAPDH) was used for normalization.

### FACS

Cells were harvested by using Accutase (Stemcell Technologies, Germany) for 5 min. Reaction was stopped using differentiation media. After centrifugation for 5 min at 3000 xg cells were fixed in 1% PFA in PBS for 15 min. Fixed cells were stored in FACS washing buffer at 4 °C. For analysis cells were incubated with primary antibodies, GFAP (1:500, rabbit IgG, DAKO, Germany or 1:100, Rat IgG, Thermo Scientific, USA), Vimentin (1:100, mouse IgG, Invitrogen, Germany), Ki67 (1:100, rabbit IgG, Santa Cruz Biotechnology, Germany) in Saponin buffer (0.5% Saponin, 0,5% BSA, 0.02% NaN3) for 2 h gently shaking. After washing cells were incubated with secondary antibodies, Alexa Fluor 488 (1:1000, goat anti-rabbit IgG), Alexa Flour 647 (1:1000, goat anti-mouse IgG) or Alexa Fluor 568 (1:1000, goat anti-rat IgG; all Molecular Probes, Germany) for 1 h gently shaking. FACSCalibur and CellQuest Pro (BD, Germany) were used for cell analysis.

### Amplex red assay

Amplex Red cholesterol assay (Molecular Probes, Germany) was used to quantify the amount of cholesterol as described recently [44–46]. Therefore, differentiated cells were washed with HBSS and harvested with Accutase (Stemcell Technologies, Germany). Enzymatic reaction was stopped with medium and cells were centrifuged at 3000 xg for 5 min. Cell pellets were resuspended in 0.1% SDS solution (in PBS) and lysis of the cells was performed by 5 freeze and thaw cycles using liquid nitrogen and tap water. Protein concentrations in lysates were measured using the bicinchoninic acid assay (Thermo Scientific, USA).

### Statistical analysis

Analysis of the data was carried out with GraphPad Prism 6 (GraphPad Software Inc., USA). Data are given as mean ± SEM. Unless otherwise stated, unpaired t-test was used to test for significance, with * = *p* < 0.05, ** = *p* < 0.01, *** = *p* < 0.001.

## Results

Gliosis is accompanied by the emergence of reactive astrocytes and can be determined not only by an increased number of GFAP positive (GFAP^+^) cells but also by the upregulation of other IFs like vimentin and nestin, as well as a rise of proliferating Ki67 positive cells. Here, we used these criteria to approve gliosis in iPSC derived glial cells. Differentiation of the progenitor cells resulted in a mixed population of glia cells and of neurons, wherein the cultures contained around 35% neurons (data not shown), independent of the genotype of the cells, but an increased glial population in the mutated cell lines. Experiments were performed with cells differentiated for 6 weeks as we found other hallmarks of NPC1 like accumulation of cholesterol or GM2 after this time of differentiation [27, 28].

### Analysis of gliosis in NPC1 patient specific iPSC derived glial cells

**Fig. 1:**
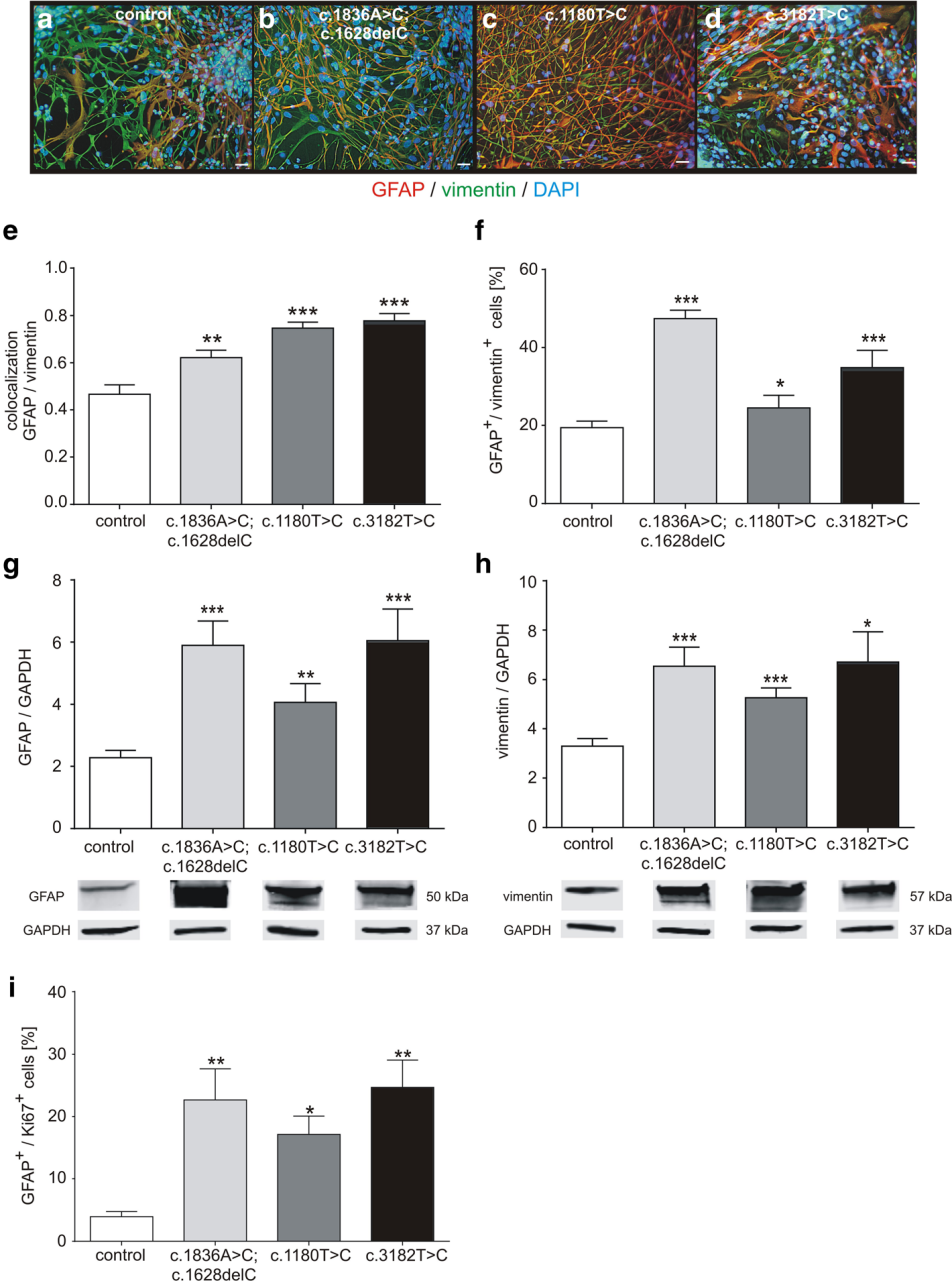
Analysis of gliosis marker. **a**-**d** *NPC1* mutant cell lines contained a higher amount of GFAP^+^ and vimentin^+^ cells (red, **a**-**d**). DAPI staining (blue) indicates nuclei. Scale 100 μm. (**e**). Colocalization analysis of GFAP and vimentin revealed a significantly increased amount of double positive cells in all *NPC1* mutant cell lines. **f** FACS analysis of GFAP^+^/vimentin^+^ cells confirmed an increased amount of glia cells in *NPC1* mutant cell lines (*N* = 5–7, *n* = 14–23). **g** In addition, semi-quantitative protein measurement by western blot demonstrated a higher amount of GFAP (*N* = 7–11, *n* = 26–43) and **h** an increased amount of vimentin in NPC1 mutant cells (*N* = 5–7, *n* = 14–23). **i** FACS analysis of cells positive for GFAP and Ki67, elucidated an increased amount of double positive cells indicating gliosis *NPC1* mutant cell lines (**g**; *N* = 3–4, *n* = 7–9)

At first we were interested in the amount of glia cells in the here used human patient-specific iPSC derived cell model. Therefore, glial fibrillary acidic protein (GFAP) and vimentin were stained (Fig. 1a-d), revealing an increased amount of GFAP positive cells (GFAP^+^), as well as vimentin positive (vimentin^+^) cells, in cell lines bearing a *NPC1* mutation. Higher coefficients of colocalization analysis confirmed this observation in *NPC1* mutant cells (Fig. 1e). In addition, flow cytometry analyses were done to quantify the proportion of GFAP^+^/vimentin^+^ cells (Fig. 1f), revealing a significantly increased amount of glial cells in all *NPC1* mutant cell lines in comparison to the control cell line after 6 weeks of differentiation. No differences between the amount of GFAP^+^ control cells after 2 and 6 weeks of differentiation were found (data not shown), as well as no differences were found between control cells and mutated cells after 2 weeks of differentiation (data not shown), indicating an onset of gliosis in the mutated cells later than 2 weeks of differentiation. However, to further affirm gliosis we determined the protein level of GFAP (Fig. 1g) and vimentin (Fig. 1h) by semi-quantitative western blot analyses, demonstrating significantly increased amounts of GFAP and vimentin. As further criteria of gliosis we proved the appearance of proliferative cells by means of a parallel staining of GFAP and Ki67 and determined the number of double positive cells by FACS analysis. This experiment revealed a significantly increased number of GFAP^+^/Ki67^+^ cells in all *NPC1* mutant cell lines in comparison to control cell line (Fig. 1i).

Taken together, these results demonstrate gliosis in NPC1 patient specific iPSC derived glial cells, proved by an increased number of proliferative glial cells and an increased number of GFAP^+^ and vimentin^+^ cells. As we have demonstrated an upregulation of vimentin in our cell model system, we speculated about an altered assembly of vimentin, as well as of GFAP, both belonging to the class of intermediate filaments type III, in the iPSC derived glial cells used here. Therefore, we analysed the assembly of GFAP and vimentin, as well as the phosphorylation status of these filaments.

### Assembly and phosphorylation of IFs in iPSC derived glial cells

**Fig. 2:**
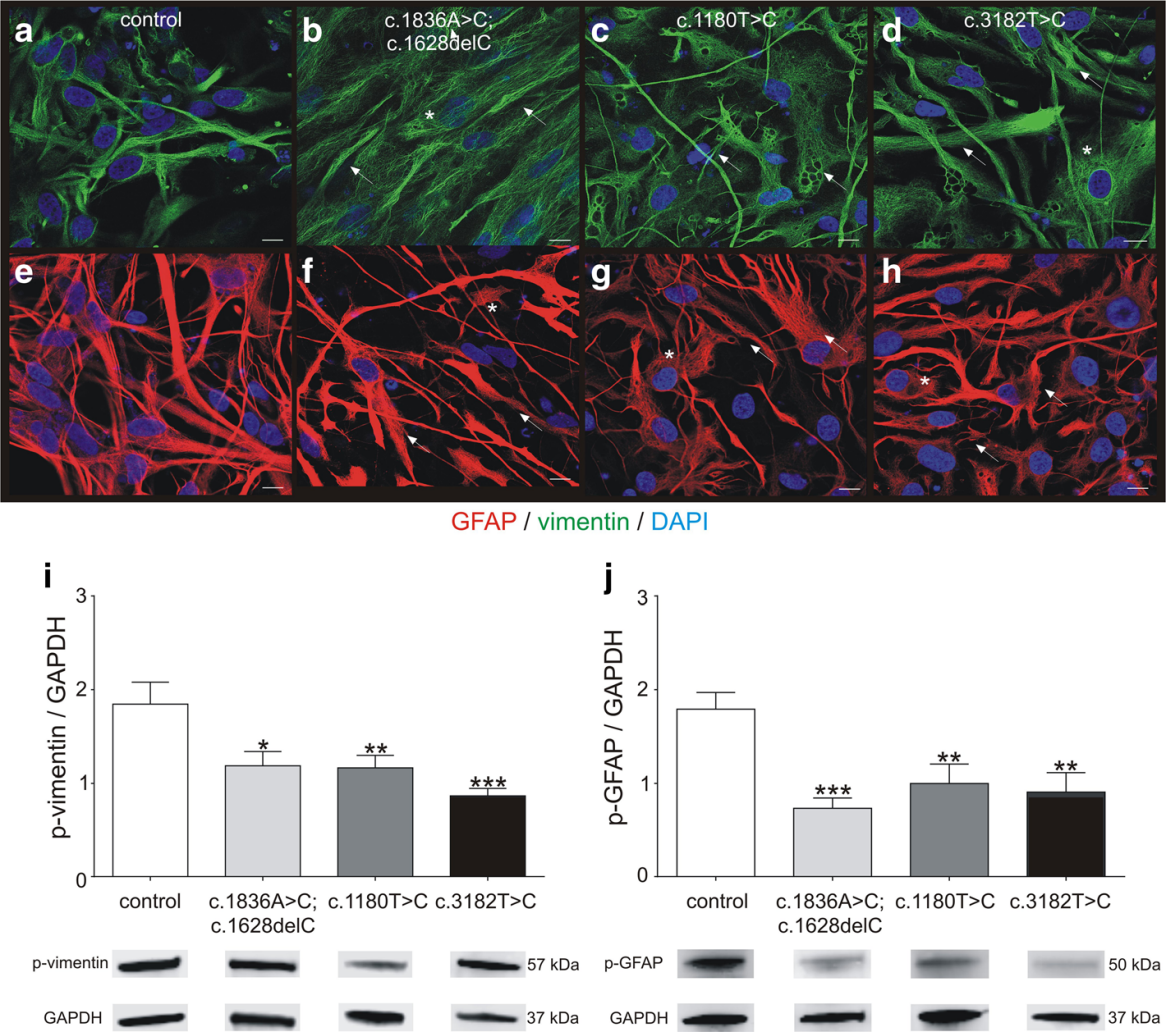
Assembly and phosphorylation of vimentin and GFAP. **a**-**d** Immunocytochemical analysis of vimentin (green) indicates altered vimentin arrangement in cells bearing a *NPC1* mutation. These cells display longer and thicker vimentin bundles (arrows), arranged in a criss-crossed manner (asterik), in comparison to control cells. **e**-**h** Similar observations were made in cells stained for GFAP red). Nuclei are stained by DAPI (blue). Scale 10 μm. **i**, **j** Amount of phosphorylated vimentin (p-vimentin) and phosphorylated GFAP (p-GFAP) was quantified by semi-quantitative western blot. All *NPC1* mutant cell lines displayed a significantly reduced amount of p-vimentin and p-GFAP indicating a hypo-phosphorylation of the IFs. (p-vimentin: *N* = 6–8, *n* = 26–40; p-GFAP: *N* = 5–7, *n* = 18–31). Examples of according western blot bands are shown below the bar graphs

Recently, it was demonstrated that the assembly of vimentin is altered on fibroblasts of NPC1 patients [37], wherein the *NPC1* mutant fibroblasts showed a disturbed arrangement of vimentin. The here used iPSC derived glial cells demonstrated comparable pattern of IF structure. Figure 2 represents immunocytochemical stainings of vimentin in control and NPC1 mutant cell lines (Fig. 2a-d). In comparison to the control cells (Fig. 2a), the NPC1 mutant cell lines revealed longer and thicker criss-crossed bundles of vimentin (Fig. 2b-d). We observed comparable changes for GFAP (Fig. 2e-h). The altered appearance is in accordance to observations in fibroblasts of NPC1 patients and indicates an altered assembly of these IFs. Next, we were interested in the phosphorylation of vimentin and GFAP as the assembly/disassembly of these IFs is regulated by phosphorylation of the IF monomers.

We used antibodies detecting the phosphorylation side serin 38 and determined the amount of phosphorylated vimentin and GFAP (p-vimentin, p-GFAP) by western blot (Fig. 2i, j). Control cells displayed the highest amount of p-vimentin and all mutant cell lines had a significantly decreased pool of p-vimentin (Fig. 2i). Comparable results were obtained for the amount of p-GFAP showing a significantly decreased amount of p-GFAP.

### Induction of gliosis by U18666A

**Fig. 3:**
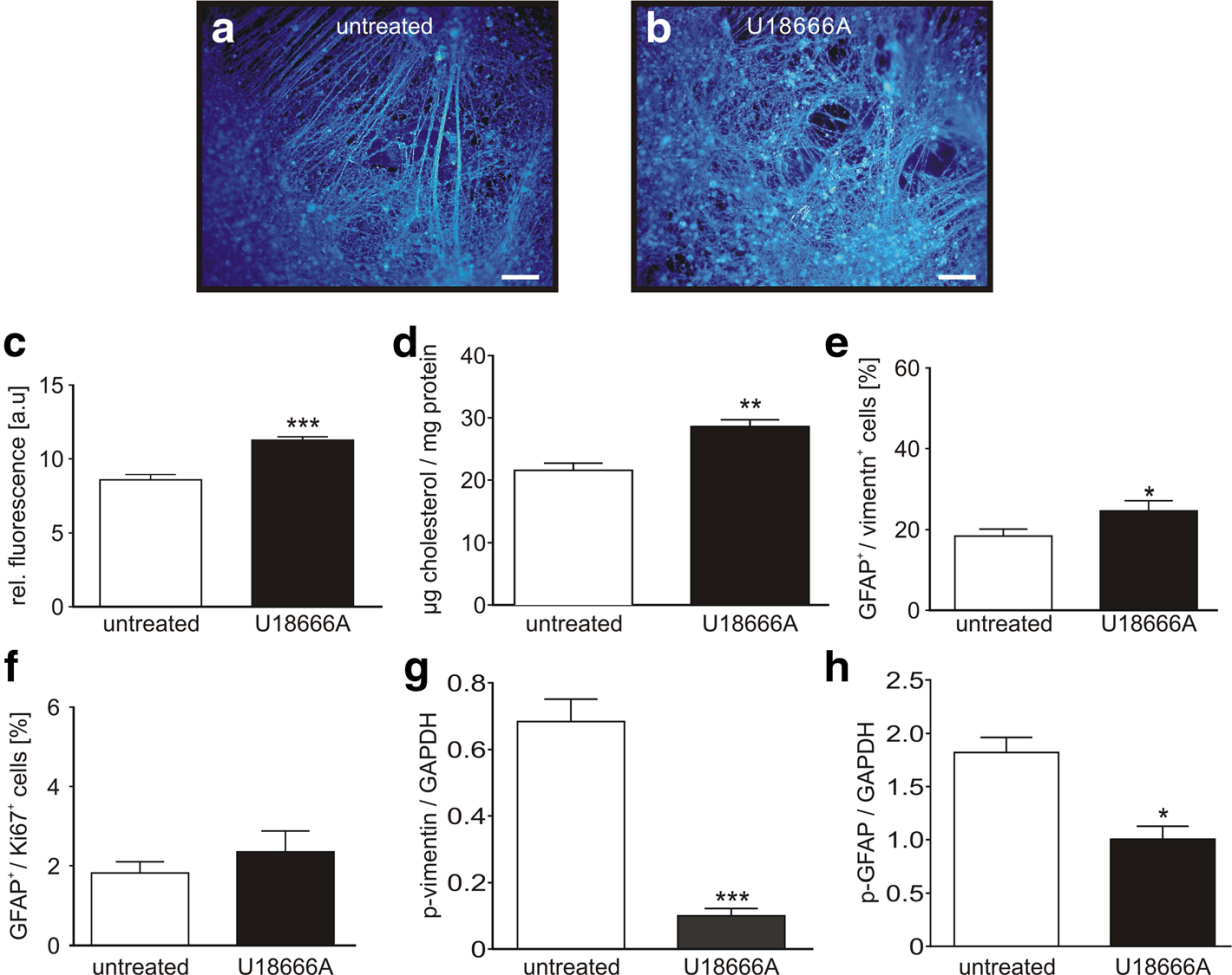
Induction of gliosis by U18666A. **a** Unaffected control cells were treated with (**b**) U18666A to induce cholesterol accumulations shown by Filipin staining (blue). Scale 100 μm. **c** Calculation of relative fluorescence confirmed higher cholesterol amount in U18666A treated cells (*N* = 4, *n* = 6–8). **d** Increased cholesterol amount in U18666A treated cells was also detected by means of the Amplex red assay (*N* = 4, *n* = 7–8). **e** FACS analysis of GFAP^+^/vimentin^+^ cells and **f** GFAP^+^/Ki67^+^ cells revealed an increased amount of reactive astrocytes (*N* = 4, *n* = 7–12). **g** Treatment with U18666A resulted in a reduced amount of p-vimentin and (**h**) p-GFAP (*N* = 4, *n* = 8–12)

U18666A is a widely used blocker of intracellular cholesterol transport, used to study the effect of induced cholesterol accumulations on cellular homeostasis [47]. Here, we used U18666A to induce cholesterol accumulations in cells of the control cell line, as we asked if this will induce gliosis. We applied U18666A (1 μg/ml) for 24 h to control cells which were differentiated for 6 weeks and analysed the above described parameters indicating gliosis. First, the application of U18666A induced clearly cholesterol accumulations, shown by Filipin staining (Fig. 3b), in contrast to untreated control cells (Fig. 3a). The determination of relative fluorescence units (Fig. 3c), as well as the quantification of cholesterol by the Amplex Red assay (Fig. 3d), demonstrated an increased amount of cholesterol in the U18666A treated cells. Moreover, we found a significantly increased amount of GFAP^+^/vimentin^+^ cells (Fig. 3e) and GFAP^+^/Ki67^+^ cells (Fig. 3f), demonstrating an U18666A dependent induction of reactive astrocytes. Accordingly to the observed hypo-phosphorylation in NPC1 deficient cells, we observed a significantly reduced amount of p-vimentin (Fig. 3g) and GFAP (Fig. 3 h) in U18666A treated cells. These results demonstrate that U1866A induces gliosis in control cells and suggests, that the accumulation of cholesterol reflects the trigger. If the accumulation of cholesterol directly induces gliosis or if gliosis is induced by the hypo-phosphorylation of intermediate filaments stays elusive.

### Rescue from gliosis and NPC1 phenotypical cholesterol accumulation

**Fig. 4:**
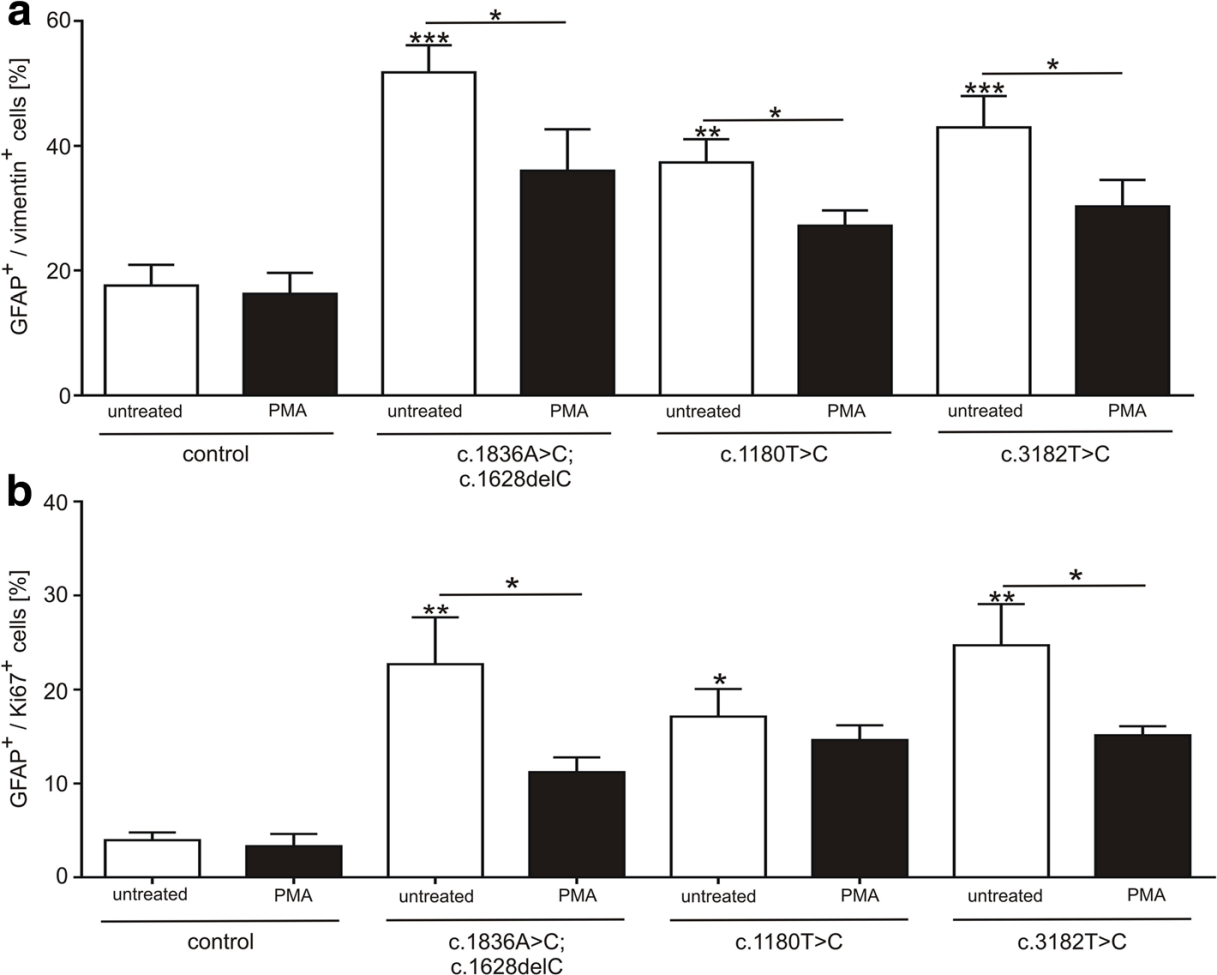
Effect of PMA on gliosis. **a** Cells were treated with 10 nM PMA and number of GFAP^+^/vimentin^+^ cells and (**b**) GFAP^+^/Ki67^+^ cells was quantified. FACS analysis revealed a significant reduction of GFAP^+^/vimentin^+^ cells in all *NPC1* mutant cell lines after PMA treatment (*N* = 4–5, *n* = 11–28). Number of GFAP^+^/Ki67^+^ cells (**b**) was reduced in all *NPC1* mutant cell lines after treatment with PMA (*N* = 4–5, *n* = 7–16). Asterisks above bars indicate significance to untreated control and asterisks above lines indicate significances between treated and untreated cells

**Fig. 5:**
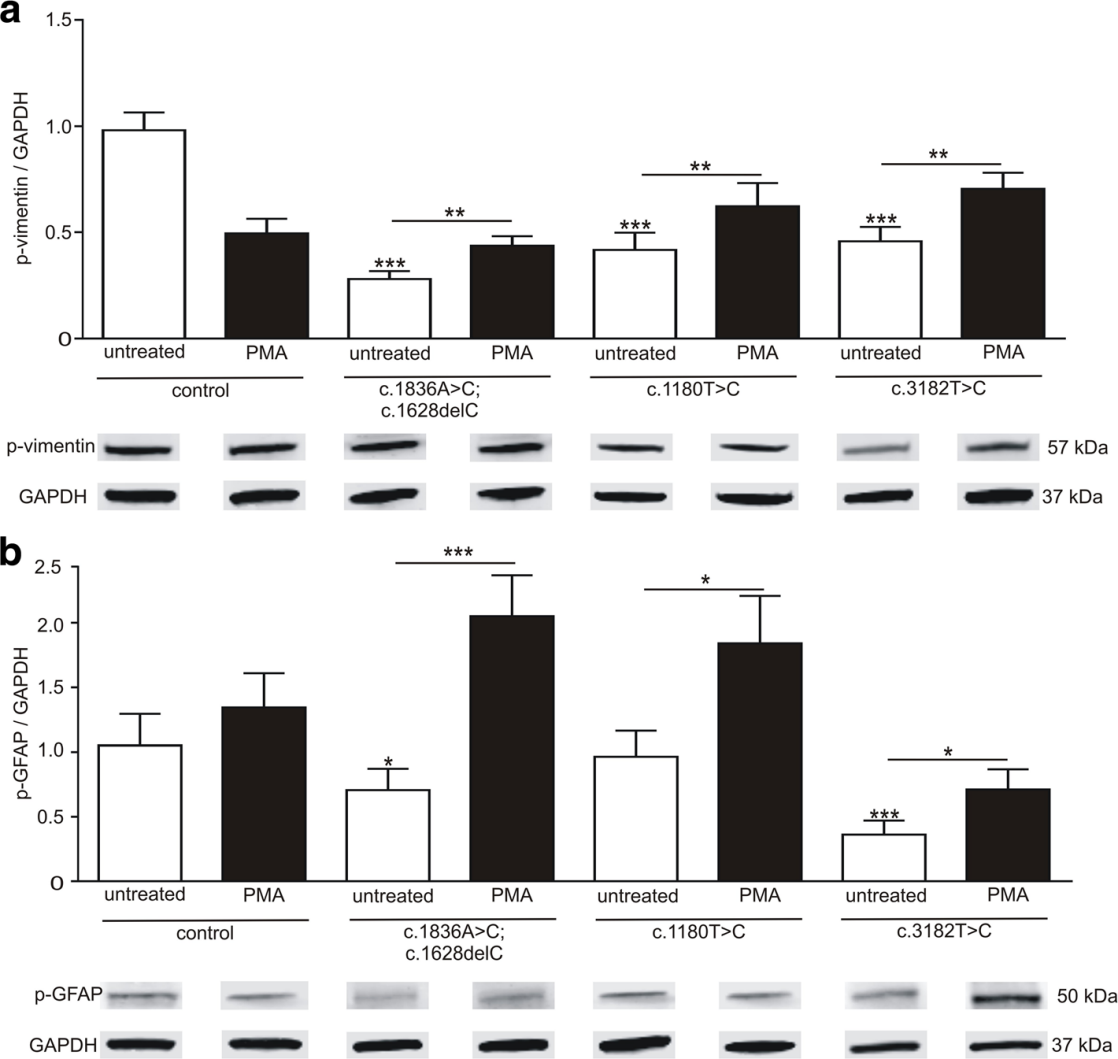
Effect of PMA on p-vimentin and p-GFAP amounts. **a** Cells were treated with 10 nM PMA and fractions of p-vimentin and **b** p-GFAP were analysed by semi-quantitative western blot. Treatment with PMA resulted in all *NPC1* mutant cell lines in significantly elevated fractions of p-vimentin (*N* = 4–5, *n* = 18–30) and p-GFAP (*N* = 4–5, *n* = 11–30). Asterisks above bars indicate significance to untreated control and asterisks above lines indicate significances between treated and untreated cells

As we have demonstrated an altered assembly of GFAP and vimentin, as well as an altered amount of p-GFAP and p-vimentin in cells carrying a NPC1 mutation and in unaffected control cells treated with U18666A, we asked next if the activation of PKC leads to a rescue of the observed gliosis as well as of the NPC1 phenotypical accumulation of cholesterol. Therefore, we treated the cells with phorbol 12-myristate 13-acetate. PMA is an activator of the protein kinase C which phosphorylates GFAP and vimentin and increases the amount of the soluble monomers. We treated the cells with 10 nM PMA for 48 h and measured the amount of GFAP^+^/vimentin^+^ cells and the amount of GFAP^+^/Ki67^+^ cells by flow cytometry (Fig. 4). The treatment of NPC1 mutant cell lines resulted in a significantly reduced number of GFAP^+^/vimentin^+^ cells (Fig. 4a), as well as a significantly reduced amount of GFAP^+^/Ki67^+^ cells (Fig. 4b), indicating a rescue of the glial cells from gliosis. To evidence the influence of PMA on the assembly cycle of intermediate filaments and the distribution of the soluble fraction, we used western blot to determine the fraction of p-vimentin (Fig. 5a) and p-GFAP (Fig. 5b). Comparable to the effect of PMA on the amount of GFAP^+^, vimentin^+^ and Ki67^+^ cells, the quantity of p-vimentin and p-GFAP increased after PMA treatment in all *NPC1* mutant cell lines, demonstrating a rescue of the disturbed intermediate filament assembly cycle.

Finally, we asked if the activation of PKC and the subsequently restoration of p-vimentin and p-GFAP had an impact on the NPC1 phenotypical cholesterol accumulation, which we described recently for these *NPC1* mutant cell lines [27, 28]. The effect of PMA on the cholesterol accumulation was assessed by Filipin staining, the quantitation of the staining by fluorescence intensities, and the total cholesterol amount by using the Amplex Red assay.

**Fig. 6:**
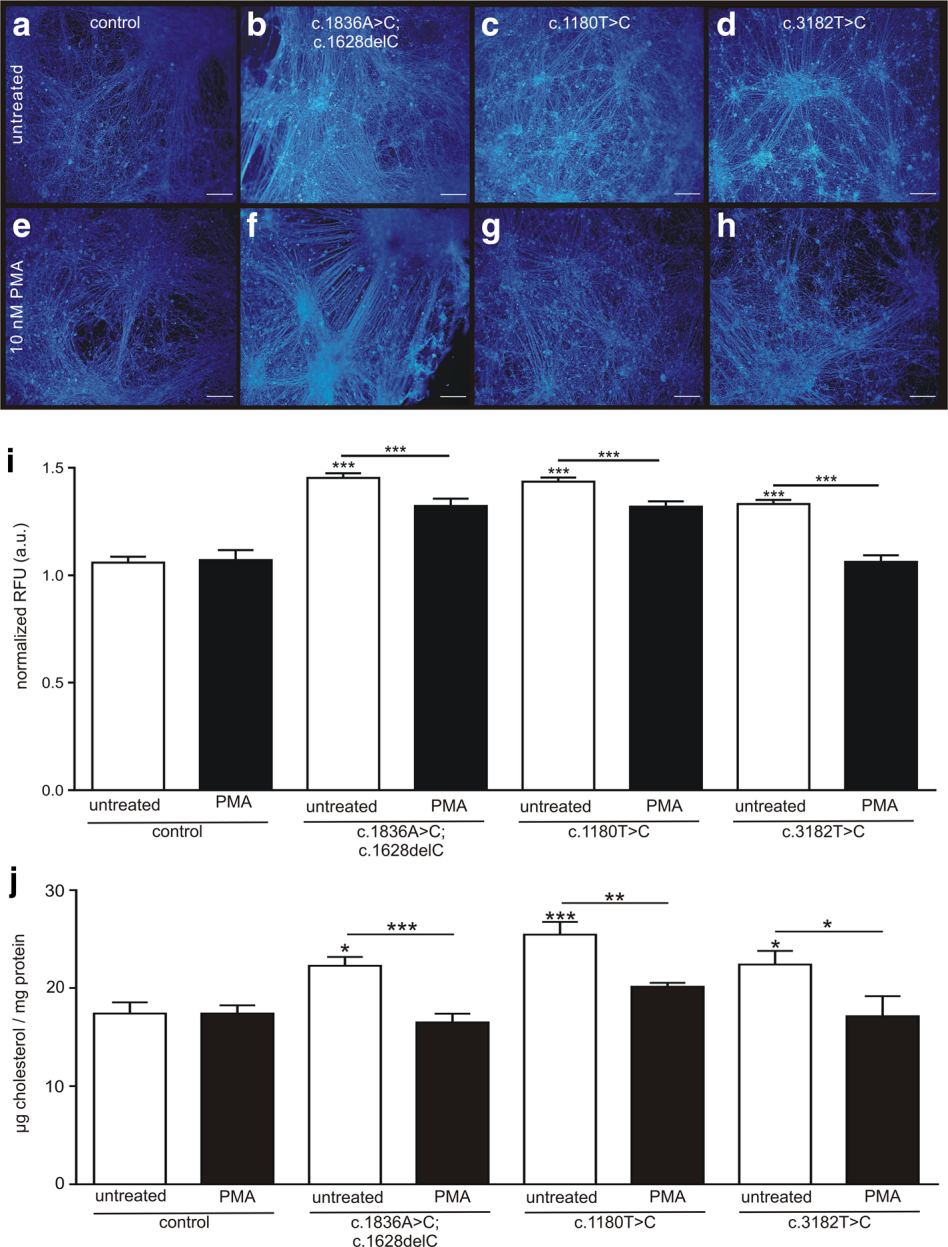
Effect of PMA on cholesterol amounts. **a**-**h** Filipin staining (blue) was used to assess cholesterol content and demonstrated typical accumulations in all *NPC1* mutant cell lines. Cholesterol accumulations were ameliorated after the treatment with PMA, resulting in a Filipin staining pattern comparable to the control cells. Scale 100 μm (**i**). Accordingly the analysis of the fluorescence intensities revealed a decreased amount of cholesterol (*N* = 4, *n* = 40–50). **j** Similar results are shown for the quantification of the cholesterol amount using the Amplex red assay. The amount decreased significantly to the cholesterol amount of control cells (*N* = 4–5, *n* = 6–18). Asterisks above bars indicate significance to untreated control and asterisks above lines indicate significances between treated and untreated cells

We observed the typical accumulation of cholesterol in the *NPC1* mutant cell lines in Filipin stainings in comparison to the control (Fig. 6a-d). Treatment of the cells with PMA (Fig. 6e-h) resulted in a decrease of the Filipin staining, at least in the cells carrying the mutations c.1180 T > C and c.3182 T > C, indicating less accumulation of cholesterol. This was confirmed by the analysis of the Filipin fluorescence signal (Fig. 6i), as well as by the Amplex Red assay (Fig. 6j). All *NPC1* mutant cell lines displayed a significantly reduced amount of cholesterol. Moreover, these amounts were not distinguishable from the cholesterol amount of the control cell line. Taken together, the activation of PKC by PMA triggered a rescue of iPSC derived glial cells from gliosis, induced an increase of phosphorylated IFs and attenuated the NPC1 phenotypical accumulation of cholesterol.

## Discussion

### Analysis of gliosis in NPC1 patient specific iPSC derived glial cells

Gliosis, the emergence of reactive astrocytes and microglia, is a universal event in the central nervous system after any kind of tissue damage and displays a physiological, normally, neuroprotective, reaction. But, in case of long lasting, chronical activation, glial cells starts to release mediators of cytotoxicity leading to a higher vulnerability of surrounding neurons or neuronal death [48]. Consequently, it stands to reason that dysregulation of normal astrocyte function contributes to the progression neurological disorders.

However, a major hallmark of gliosis is an increased amount of glia cells, especially reactive astrocytes, which upregulate GFAP and vimentin and re-express nestin [49]. Consequently, gliosis can be elucidated by an increased number of GFAP positive cells [16] and an upregulation of the IFs vimentin and nestin, as well as an increased number of proliferative cells, demonstrated by Ki67 expression or BrDU incooperation. In regards of NPC1, gliosis, as well as marker for neuroinflammation, were shown in human post mortem brain biopsy material in several regions of the brain and in murine models of NPC1 [24–26]. An increased number of reactive astrocytes and abnormal morphological changes are described in the broadly used BALB/c_Nctr-Npc1m1N/−J NPC1-deficient mouse strain [20, 21, 50]. NPC1-deficient mice revealed an upregulation of glia cells after 4 weeks of age and astrocytes showed an atypical morphology by less elaborated processes and swollen cell bodies [23]. In accordance to findings in murine models, we observed higher amounts of GFAP^+^/vimentin^+^ cells, as well as an increased protein amount of both IFs in our human disease model system. Cells double positive for GFAP and Ki67^+^ reflect proliferative reactive astrocytes in NPC1 mutational cell lines, displaying a further characteristic of gliosis. Thus, we conclude that glial cells derived from NPC1 patient-specific iPS cells undergo gliosis, representing a further hallmark observed during the progression of NPC1. We emphasize that gliosis was not induced, but displays an intrinsic feature of NPC1 deficient cells of this model system. Until now, studies analyzing gliosis in human in vitro cell model systems are missing and to our knowledge this is the first study demonstrating gliosis in a human iPSC based cell model.

Beyond the question if iPSC derived glial cells undergo gliosis, we were interested in the assembly of the GFAP and vimentin as it was recently described that NPC1-patient derived fibroblasts display hypo-phosphorylation of vimentin [37]. While vimentin is downregulated during brain development it is upregulated in astrocytes undergoing gliosis [16]. Nevertheless, vimentin is discussed to be also upregulated as a damage-response mechanism in neurons in neurodegenerative disease like Huntington or Alzheimer disease, whereby vimentin is involved in neurite extension and synaptic recovery [51]. This aspect merits further studies of the function of vimentin in neurodegenerative diseases, to elucidate the contribution of IFs to the progression or attenuation of disease emergence.

### Assembly and phosphorylation of IFs in iPSC derived glial cells

In regards of the assembly of the IFs GFAP and vimentin, we observed changes in the appearance in NPC1 mutant cell lines. In immunocytochemical stainings GFAP and vimentin appeared in more densely packed aggregates, organized in a criss-cross manner within the cytosol. This appearance is in accordance with stainings of vimentin in fibroblasts of NPC1 patients [37] and indicates a disturbed assembly/disassembly cycle for vimentin and GFAP. Consistently, we found significantly decreased amounts of the phosphorylated, soluble, forms of GFAP and vimentin in *NPC1* mutant cell lines, in accordance to studies performed with fibroblasts of NPC1 patients [37, 38]. These studies demonstrated not only a disturbance of vimentin assembly, but vimentin was found to interact with Rab9, during lipid movement from late endosomes, and Rab7a, involved in vesicular membrane trafficking [52]. This interaction is altered in NPC1 disease due to lipid and cholesterol accumulation in late endosomes resulting in an inhibition of PKC, hypo-phosphorylation of vimentin and endosomal dysfunction. Hypo-phosphorylation of vimentin leads to aggregation and enclosure of Rab9, finally resulting in transport deficiencies and blocked lipid egress [37, 38]. Most recently, vimentin aggregates were shown to inhibit trafficking of mitochondria in giant axonal neuropathy [53] strengthening the hypothesis that reduced phosphorylation of intermediate filaments leads to a trafficking defect in neurodegenerative disease. These alterations in IF type III are assumed to inhibit lysosomal exocytosis [37, 38] which is a known mechanism for NPC1 mutational cells to release stored cholesterol. For instance, cholesterol lysosomal exocytosis could be enhanced by HPB-cyclodextrin [54] and δ-tocopherol [55]. A possible mode of action of these substances could be the induction of a calcium-influx which in turn activates PKC, subsequently leading to a phosphorylation of IFs, initiating the lysosomal exocytosis leading to a depletion of cholesterol in *NPC1* mutant cells.

### Rescue from gliosis and NPC1 phenotypical cholesterol accumulation

As an inhibition of PKC, by lysosomal lipid accumulation, was demonstrated in fibroblasts of NPC1 patients [37, 38] and PKC activation restores subcellular cholesterol transport in NPC1-deficient fibroblasts [38], we asked if an activation of PKC ameliorates the features of gliosis, hypo-phosphorylation of IFs, and finally the accumulation of cholesterol observed in iPSC derived glial cells. Thus, we treated the cell cultures with the PKC activator PMA and determined marker for gliosis, amount of phosphorylated IFs, and cholesterol. In regards of gliosis, PMA treated cell cultures demonstrated a reduced number of GFAP^+^/vimentin^+^ cells, as well as reduced number of GFAP^+/^Ki67^+^ cells, revealing an amelioration of gliosis.

In accordance to the study of Walter and coworker [37], the treatment with PMA increased the phosphorylated amount of vimentin. In addition to this study, we analysed glial cells and consequently GFAP and vimentin and found a similar impact of PMA on GFAP. This was to be expected as GFAP and vimentin belong both to the family of intermediate filaments type III. The cascade leading to a reduced amount of phosphorylated GFAP stays elusive, but we speculate that the suggested interrelationship of PKC activation, vimentin and Rab9 [37] can be adapted to GFAP. Following this model, the lysosomal accumulation of cholesterol would be the starting point of this vicious circle.

In support of this hypothesis we used U18666A to induce an accumulation of cholesterol in control cells. The treatment with U18666A resulted in an accumulation of cholesterol as expected, but moreover we observed gliosis in the control cells, demonstrated by a significantly increased number of GFAP^+^/vimentin^+^ cells, accompanied by a reduced amount of phosphorylated GFAP and vimentin. Recent studies described U1866A induced cholesterol accumulations in rat astrocytes influencing the metabolic pathway of these cells [56, 57], but effects in regards of gliosis were not topic of these studies.

Moreover, we observed a strong impact of the PMA treatment on the cholesterol amount in NPC1 mutant cells. As a hallmark of NPC1, the here used NPC1 mutant cell lines [27, 28], as well as other iPSC derived NPC1 neuronal cells [29–31] showed significant cholesterol accumulations. PMA induced a reduction of cholesterol accumulations, demonstrated by Filipin stainings and by Amplex Red Assay, where latter one elucidated a normalization of the cholesterol amount, comparable to the cholesterol amount of control cells. In accordance to our results, a redistribution of cholesterol upon the activation of PKC was recently demonstrated in NPC1-deficnet fibroblasts [38].

Bringing together our results and the results obtained from NPC1-deficient fibroblasts [37, 38], we conclude that the accumulation of cholesterol initiates a perturbation of PKC signaling, leading to altered assembly of IFs. We speculate, that this cascade contributes to the initiation of gliosis, observed in NPC1 mutant cell lines as well as in U18666A treated control cells. Consequently, gliosis appears to be primary effect of the cholesterol accumulation within glial cells and not a secondary effect mediated by the loss of neurons in NPC1. Actually, the contribution of gliosis to the progression and/or phenotypical occurrence of NPC1 is controversially discussed. One certain feature of NPC1 is an activation of astrocytes, observed both in mouse models and NPC1 patients [58]. But, the contribution of astrocytes to the neurodegenerative processes is controversially discussed. Knock-down experiments of *NPC1*, restricted to astrocytes or neurons in the CNS of mice hint at astrogliosis as a secondary process with a low impact on the disease progression [59–62]. On the other hand, severe symptoms of murine NPC1-models were significantly ameliorated upon the astrocyte-specific knock-in of *NPC1* [63, 64]. Still, an almost complete recovery was only achieved by double knock-in of *NPC1* into astrocytes and neurons [65], indicating a cooperative mechanism underlying the recovery. If an impaired signaling in astrocytes, based on a corrupted PKC signaling and thus corrupted PKC dependent processes, ends up in a deleterious vicious circle leading to cell loss is speculative and stays enigmatic, but merits further studies regarding the contribution of glial cells and gliosis to the pathogenic mechanism underlying NPC1, wherein patient specific iPSC models provide promising tools in regards of disease modelling.

## Conclusion

Here we demonstrated gliosis in a cell model system based on NPC1 patient specific iPS cells. We found that gliosis is an intrinsic feature of this model system, reflecting one of the pathological distinguishing marks of NPC1. For sure, this feature provides the opportunity to study the impact of gliosis as well as the interplay between glial cells and neurons on the pathogenic mechanisms of NPC1 in a human model system. Besides the applicability of these model system in disease modelling of NPC1, we described the alterations of intermediate filaments in regards of structural features and regulation by PKC. More importantly, we confirmed an impact of PKC signaling on the pathogenesis of NPC1, presenting the possibility to develop new intervention strategies to ameliorate the progression of NPC1.

## Abbreviations

DMEM: Dulbecco’s Modified Eagle’s Media
GAPDH: Glyceraldehyde 3-phosphate dehydrogenase
GFAP: Glial fibrillary acidic protein
GFAP^+^: GFAP positive cells
IFs: Intermediate filaments
NPC1: Niemann-Pick disease Type C1
PBS: Phosphate Buffered Saline
PFA: Paraformaldehyde
p-GFAP: Phosphorylated GFAP
PKC: Protein kinase C
PMA: Phorbol 12-myristate 13-acetate
p-vimentin: Phosphorylated vimentin
vimentin^+^: Vimentin positive cells
